# Males as victims of intimate partner violence — results from a clinical-forensic medical examination centre

**DOI:** 10.1007/s00414-021-02615-x

**Published:** 2021-04-29

**Authors:** Xenia Wörmann, Sandra Wilmes, Dragana Seifert, Sven Anders

**Affiliations:** Department of Legal Medicine, University Medical Center Hamburg-Eppendorf, Butenfeld 34, 22529 Hamburg, Germany

**Keywords:** Intimate partner violence, Domestic violence, Male victimisation, Clinical-forensic medicine, Gender differences

## Abstract

Only few studies have reported on males as victims of intimate partner violence (IPV) so far. The aim of the present study is to analyse frequency and case characteristics of physical violence against male IPV victims examined in a clinical-forensic medical examination centre for victims of violence in Germany over an 11-year period, contributing to a better understanding of IPV in men. Male victims represented 6.2% of IPV cases (*n* = 167) with a median age of 40 years. Cases were reported to the police in 78.4% before medicolegal examination. In 60.5% of the cases, the perpetrator was the current partner, and 82% occurred in a domestic environment with a predominance of female offenders. In more than half of the cases (57.5%), the victims consulted the examination centre without prior healthcare utilisation. About one-third of the victims reported previous IPV (31.7%). The findings point to the relevance of men as victims of IPV, case group–specific risk factors, injury-dependent behaviour related to healthcare utilisation, the need to establish or strengthen specialised support services for affected men and underscore the importance of clinical-forensic services in documenting and assessing violence-related injuries.

## Introduction

Intimate partner violence (IPV) refers to any type of physical, sexual or psychological violence, including stalking, by a current or former partner [1]. IPV is a global burden that affects all social classes and has recently received renewed media attention during the current COVID-19 pandemic with restrictions in social and public life, as an increase in IPV has been reported in several studies [2–5]. IPV affects both females and males. The Mankind Initiative, an organisation for male victims of IPV in the UK, noticed a 35% increase in phone calls during the lockdown period [6].

In Germany, about 17% of all recorded crimes are related to IPV with a total number of 141.792 victims in 2019 [7]. Between 2017 and 2019, total numbers of IPV increased by 2.08%, while the number of male victims increased by 7.8%, representing 19% of IPV cases in 2019 [7–9]. A quantitative survey on physical violence and forced sexual acts against men reported a lifetime prevalence of 22.6% with a quarter being injured and none calling the police [10]. Further studies showed a prevalence of 4.0–11.0% within the previous year and a prevalence up to 20.3% in the current relationship [11–14]. Reports on medicolegal characteristics of male victims of IPV in Germany are scarce. A recent German study reported on IPV injury patterns of 16 men examined over a 6-year period [15, 16], while a portion of 11–12.8% of males among clinical-forensic examinations of IPV victims was observed in other European countries [17–19].

IPV associated injuries have been shown to occur most frequently in the neck, head and face regions [20], predominantly being minor injuries like scratches and hematomas resulting from non-instrumental blunt force trauma [17–19, 21], while instrumental violence (e.g., household items, weapons) has been reported to occur less frequently [17, 21]. Research on gender differences in IPV showed that female perpetrators were more likely to hit, bite, kick and use objects to hit or throw, whereas men more often beat, choke or strangle the victim [22].

Both victim and perpetrator characteristics and risk factors can serve as a starting point for the development of appropriate programmes and counselling. Among risk factors for IPV victimisation are witnessing IPV in childhood, short-term relationships, young age and alcohol abuse [12, 13, 23–26]. Besides public campaigns, training of healthcare professionals, law enforcement agencies and educators are important for both prevention and diagnosis of IPV. However, most IPV victim support centres in Germany offer services to female victims exclusively, therefore specific offers for affected males would be highly eligible [16].

The aim of the present study is to analyse frequency and case characteristics of physical violence against male IPV victims examined in a clinical-forensic medical examination centre for victims of violence in Germany, contributing to a better understanding of IPV in men in order to help establishing appropriate future victim support programmes and counselling.

## Methods

The Forensic Medical Examination Centre for Victims of Violence at the Department of Legal Medicine of the University Medical Centre Hamburg-Eppendorf (UKE, Hamburg, Germany) allows examinations for law enforcement agencies and provides a low-threshold access for victims of violence independent of filing a police report. Independent of access pathway, a whole-body medicolegal examination, documentation of injuries and medicolegal expert opinion are provided. In addition, a standardised data sheet is filled in for every case and data are transferred to an electronic in-house database. Included in this study are male adult victims of IPV examined at the Forensic Medical Examination Centre during an 11-year period from January 2006 to December 2016 Inclusion criteria were male gender, age 18 years and older and a current or former partnership with the perpetrator. For identifying appropriate cases, a search was conducted using the in-house database based on Microsoft Access (Microsoft Corporation, Albuquerque, NM, USA). The following filters were applied for the search according to the in-house database categories: male victim, domestic violence and exclude self-injuries. The initial search led to identification of 181 cases. For all cases, original files were reviewed and analysed for the study. During data analysis, a total of 14 cases was excluded from the study (age under 18 years, perpetrator other than partner or former partner), resulting in 167 included cases. For comparison of the male cases to the total number of examined IPV cases during the study period, an additional search in the in-house database was performed using the following filters: female victim, domestic violence, exclude self-injuries, age 18 years and older.

File reports of male cases were analysed according to the following criteria; results were recorded anonymized using Microsoft Excel (Microsoft Corporation, Albuquerque, NM, USA):

- (a) Victim and perpetrator characteristics: age of victim at the time of consultation, gender of perpetrator, number of perpetrators
- (b) Victim-perpetrator relationship: relationship status, shared or separate homes, report of previous IPV
- (c) Institutional support: healthcare utilisation prior to medicolegal examination, repeated consultation during study period, filing of a police report
- (d) Characteristics of the violent attack: reported manner of violence/mechanism of the injury, locations and types of injuries, in case of instrumental violence: kind of object or weapon, weekday and place of attack

Missing data were excluded from further analysis. Descriptive statistical analysis was performed using SPSS (Statistical Package for Social Science; IBM®) version 23. Fisher’s exact test was used to compare qualitative variables, a *p* value of < 0.05 was considered statistically significant. For graphic illustrations, Microsoft Excel and Affinity Designer version 1.9.1 (Serif Ltd., Nottingham, UK) were used.

## Results

**Fig. 1:**
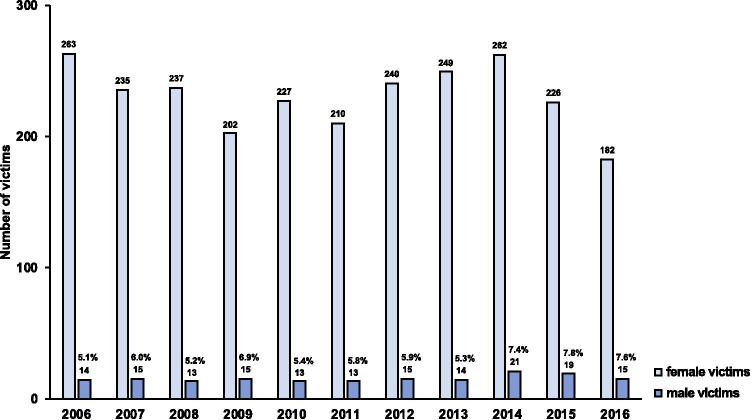
Numbers of male and female IPV victims at the Forensic Medical Examination Centre for Victims, Hamburg, Germany

From 2006 to 2016, a total of 2714 cases of domestic violence were examined at the Department of Legal Medicine in Hamburg, among them 2533 female and 181 male victims. For male victims, 167 cases met the inclusion criteria for further analyses. Male victims represented 5.1 to 7.8% of IPV cases per year during the study period, showing a slightly upward trend in the last 3 years analysed (Fig. 1; mean 6.2%; SD ± 1.0).

### Victim and perpetrator characteristics, previous IPV and care pathway

Age span of male victims was 19 to 90 years (Median 40 years, mean 40.6 years; Table 1). Cases were reported to the police in 78.4% at the time point of the medicolegal examination. In 60.5% of the cases, the perpetrator was the current partner (former partner: 39.5%) and in about half of the cases the victim and the perpetrator shared the same apartment (53.9%). About one-third of the victims reported previous IPV (31.7%). In 14 of these 53 cases, older injuries or scars were present upon medicolegal examination, representing signs of previous IPV. During the study period, three victims (1.8%) consulted the Forensic Medical Examination Centre repeatedly due to offences by their intimate partner. Perpetrators were predominantly female (91.6%). In the majority of cases, the victims reported to be attacked by a single perpetrator (92.8%). If more than one perpetrator was reported, 62.5% of these cases involved family members of the current or former partner: children (*n* = 2), parents (*n* = 5) or the perpetrator’s new partner (*n* = 3). In one case, the victim identified a friend of the perpetrator; in two cases, the person was unknown to the victim and in three further cases, it was not possible for the victim to clearly identify the offender.

**Table 1:** Victim and perpetrator characteristics and healthcare utilisation in cases of IPV against men (*n.s.* not specified)

			*n* = 167
Victims	Age	Median Mean, SD Min Max	40 y 40.6 y, ± 12.5 19 y 90 y
	Filing a police report	Yes	78.4% (131)
		No	21.6% (36)
Perpetrators	Gender	Female	91.6% (153)
		Male	8.4% (14)
	Number of perpetrators	1	92.8% (155)
		> 1	7.2% (12)
	Relationship	Current partner	60.5% (101)
		Former partner	39.5% (66)
	Shared apartment	Yes	53.9% (90)
		No	43.7% (73)
		n.s	2.4% (4)
Previous IPV	Report of previous IPV	Yes	31.7% (53)
		No	66.5% (111)
		n.s	1.8% (3)
	Scars, injuries of previous IPV	Yes	8.4% (14)
		No	90.4% (151)
		n.s	1.2% (2)
	Repeated consultation of victim during period of evaluation	Yes	1.8% (3)
		No	98.2% (164)
Healthcare utilisation	First contact to healthcare system	Forensic medical examination Centre for victims Emergency department Office-based physician	57.5% (96) 35.9% (60) 6.6% (11)

In more than half of the cases (57.5%), the victims consulted the Forensic Medical Examination Centre without prior healthcare utilisation. In 35.9%, victims visited a hospital emergency department and 6.6% visited an office-based physician before medicolegal examination.

### Manner of physical violence

According to the victims’ reports, non-instrumental physical violence occurred in more than half of the cases (55.7%; Table 2), while instrumental violence or a combination of non-instrumental and instrumental violence were reported less frequently (17.4%, 26.3%; overall 43.7%). In cases of instrumental violence, random household objects were used twice as often (65.8%) as weapons or a combination of both (20.5%, 6.8%). Weapons were represented almost exclusively by knives (18/20 cases); in single cases, a firearm and a taser were used. During full-body examination, injuries were visible in 94.6% of the victims. Nevertheless, nine victims reported IPV, but no injuries could be found upon medicolegal examination. In accordance with the reported manner of violence, injuries due to blunt force were diagnosed in 79.0% and sharp force injuries or a combination of blunt and sharp force was found in 12.0%. In a minority of cases, thermal violence (boiling water, hot wax or electric injury), gun violence and chemical violence (cleaning agent) were diagnosed. Sexual violence was reported by two victims (1.2%). In one case, the male victim was beaten, tied up and raped twice by his former male partner (anal intercourse); in the other case, the man reported that he was beaten, followed by unwanted oral intercourse by his female partner.

**Table 2:** Characteristics of violence in cases of IPV against men (*n.s.* not specified)

		*n* = 167
Sexual violence	Denied Reported	98.8% (165) 1.2% (2)
Reported violence	Non-instrumental violence Instrumental violence Non-instrumental + instrumental violence n.s	55.7% (93) 17.4% (29) 26.3% (44) 0.6% (1)
Injuries caused by	Blunt force Sharp force Blunt + sharp force Blunt or sharp force in combination with/or thermal, chemical, gun violence No visible injuries	79.0% (132) 2.4% (4) 9.6% (16) 3.6% (6) 5.4% (9)
Instrumentalities (*n* = 73)	Random object Weapon Random object + weapon n.s	65.8% (48) 20.5% (15) 6.8% (5) 6.8% (5)

### Localisation of blunt and sharp force injuries

Blunt force injuries were found on the upper half of the body three times more often (*n* = 330; skull, face, neck, chest, upper extremities) than on the lower half (*n* = 100; abdomen, back, genital and gluteal region, lower extremities) with most injuries located on the arms (40.0%), followed by face and neck regions (14.9%; 10.9%), chest (7.7%) and skull (3.2%). Injuries on the lower half of the body were mostly located on the legs (12.6%) and back (7.2%), infrequently in the abdominal region (2.8%) and genital (0.2%) or gluteal region (0.5%). In 71.3% of cases, more than one body region was affected with a maximum of nine regions.

Sharp force injuries were diagnosed twice as often on the upper body half (*n* = 28) as on the lower half (*n* = 12), predominantly on the arms (35%), chest (12.5%), face and neck regions (10% each) and skull (2.5%). On the lower half of the body, 10% of injuries were found on the back and on the abdomen, respectively, less frequent on the legs (7.5%) and the gluteal region (2.5%). Injuries to more than one body region were seen in 28.6% (*n* = 6) with a maximum of six regions.

### Specific injuries

Scratches, predominantly caused by fingernails, were the most frequent specific injuries diagnosed (*n* = 67) followed by bite marks (*n* = 29) and grip marks (*n* = 12). In 8 cases, the violent attack had led to bone fractures; in single cases, restraint marks (*n* = 2) and sole patterns (*n* = 3) were detected. In 13 cases, victims reported on manual strangulation resulting in difficulties in swallowing or hoarseness in five cases, while none of the victims reported on unconsciousness.

### Place and time of event

In 82.0%, IPV occurred in a domestic environment: either the shared home (51.5%), the victims’ home (19.1%) or the perpetrators’ home (11.4%). In only 6% (*n* = 10), the event occurred in public spaces; single cases took place in private cars or the home of third parties (2.4%), at multiple places (1.8%) or could not be identified (7.8%). There was a slight preponderance for weekend-days with 52.1% of cases (Friday until Sunday) compared to weekdays (Monday until Thursday) with 43.7%. Rarely, multiple days of offences were reported (2.4%) or the day of the week was not documented (1.8%).

### Gender of the perpetrator and injury pattern

Despite the low number of cases in which a male perpetrator was reported, statistics pointed at some possible relationships between perpetrators’ gender and injury pattern: Injuries in the gluteal region were only present in two cases with a reported male perpetrator (*p* = 0.007); both victims denied involvement of sexual violence. Manual strangulation was more frequently reported for attacks by male offenders (21.4% vs. 6.5%; *p* = n.s). In contrast, scratch marks (41.8% vs. 21.4%), and bite marks (18.8% vs. 7.1%) were found more often upon medicolegal examination when a female perpetrator was involved (*p* = n.s.).

### Relationship status, crime scene and violence used

If IPV occurred in an ongoing relationship, victims reported a shared home to be the scene of events in the majority of cases (74.3%; Table 3). Interestingly, we could detect differences between case groups regarding the relationship status at the time point of the offence. IPV occurred more often at the victims’ apartment than at the perpetrators’ apartment when former partners were reported as offenders and persons lived in separate homes (36.4% vs. 21.2%). Furthermore, instrumental violence was reported nearly twice as often in ongoing relationships, compared to separated partnerships (54.5% vs. 27.3%). Accordingly, non-instrumental violence was observed more often with former partners as perpetrators (45.5% vs. 71.2%), and sharp force injuries occurred more often in ongoing relationships (16.9% vs. 4.5%). Group comparisons revealed significant results for differences in the location of the offence (*p* < 0.001; Table 3), instrumental vs. non-instrumental violence (*p* = 0.002) and the type of injuries (*p* = 0.018) between offences by a current or former partner.

**Table 3:** Differences among case characteristics in cases involving current or former partner offenders and results of statistical group comparison (*n.s.* not specified)

Crime scene	Current partner (*n* = 101)	Former partner (*n* = 66)	Fisher’s exact test
Shared apartment Victim’s apartment Perpetrator’s apartment Public space Others (private car, home of third parties)	74.3% (75) 7.9% (8) 4.9% (5) 4.0% (4) 3.0% (3)	16.7% (11) 36.4% (24) 21.2% (14) 9.1% (6) 1.5% (1)	*p* < 0.001
Several places of crime n.s	1.0% (1) 4.9% (5)	3.0% (2) 12.1% (8)	
Reported violence	Current partner (*n* = 101)	Former partner (*n* = 66)	
Non-instrumental violence Instrumental violence Non-instrumental + instrumental violence	45.5% (46) 22.8% (23) 31.7% (32)	71.2% (47) 9.1% (6) 18.2% (12)	*p* = 0.002
n.s		1.5% (1)	
Instrumentalities in case of instrumental violence	Current partner (*n* = 55)	Former partner (*n* = 18)	
Random object Weapon Random object + weapon n.s	65.4% (36) 21.8% (12) 7.3% (4) 5.5% (3)	66.7% (12) 16.7% (3) 5.5% (1) 11.1% (2)	*p* = not significant
Injuries caused by	Current partner (*n* = 101)	Former partner (*n* = 66)	
Blunt force Sharp force Blunt + sharp force Other (thermal, chemical, gun violence exclusively or in combination with sharp and/or blunt violence, no injuries)	75.2% (76) 2.0% (2) 14.9% (15) 7.9% (8)	84.9% (56) 3.0% (2) 1.5% (1) 10.6% (7)	*p* = 0.018

### Police involvement and healthcare utilisation

In 78.4% of all cases, the victims had filed a police report before the time of the forensic examination. However, a comparison between the case groups revealed significant differences: All victims of sharp violent injuries had filed a police report, compared to 75.3% in the other case groups (*p* = 0.008). In contrast, victims who had only suffered blunt force injuries had called the police less often (74.2%) than the other case groups (94.3%; *p* = 0.01).

Comparable results were found for healthcare utilisation prior to medicolegal examination. While victims with sharp force injuries sought emergency department care in 76.2%, the remaining case groups did seek for medical help less frequently (emergency department: 30.2%, office-based physician: 7.5%; *p* < 0.001). Accordingly, 63.6% of victims with blunt force injuries did not visit an emergency department or an office-based physician (28.8%, 7.6%) before medicolegal documentation of injuries, which was significantly less frequent compared to the other case groups (62.8%, 2.9%; *p* = 0.001).

## Discussion

With a prevalence of 6.2% among IPV cases over the 11-year study period, our study emphasises the relevance of male victims of IPV. Nevertheless, this rate is lower than numbers given in the German official annual crime statistics, which revealed a frequency of about 18% during the period of evaluation [27, 28], and comparable evaluation data from European forensic centres range between 9.8 and 13.0% males among IPV victims [17–19]. The preponderance of female victims of IPV visiting forensic medical examination centres is attributed not only to a higher number, but also to a higher likelihood among women to report incidents of violence to formal institutions, as they are better informed by a greater number of support services targeting women [19]. Accordingly, it has been discussed that male victims underreport violent offences due to a sense of shame and fear, injuries considered to be “minor” and a lack of information and appropriate support [14, 29].

In the present study, 78.4% of victims filed a police report prior to medicolegal examination, whereas lower rates were reported by other workgroups [17]. Furthermore, our data revealed significant differences between case groups regarding the manner of inflicted injury with lower numbers of police involvement following blunt force compared to sharp force attacks. This underlines the assumption that underreporting might be associated with “minor” injuries.

The proportions of current or former partners as perpetrators and the observation that a shared household is the predominantly reported crime scene are consistent with the findings of previous studies and have been reported for both male and female victims of IPV [17–19]. Interestingly, statistical group comparison revealed significant differences between case groups (Table 3). Regarding the scene of events, the results point at a higher risk for victimisation in the victims’ apartment than in the perpetrators apartment in case the offender was a former partner. We believe that these findings are of high relevance for counselling male individuals who are living in relationships high at risk for the occurrence of IPV, as offences in public are rare events, whereas domesticity might be an unsafe place. While individuals living in an ongoing partnership in a shared home often have no alternative location in the event of conflict, separated partners should be advised to meet in public rather than at home, if the risk of IPV is evident. On the other hand, we observed a significantly higher number of instrumental violence in ongoing compared to separated partnerships. This implies a higher risk of more serious injuries and necessity of awareness for the potential danger of upcoming situations that might result in IPV, issues that should be covered by support services when counselling male individuals. These findings and the observation that events showed a preponderance for weekend-days support the fact that IPV occurs shielded from public view in close social situations. When several perpetrators were involved, these were predominantly persons from the close social setting of the perpetrator and victim, underlining the possible influence of the social framework on IPV.

In our study, 8.4% of perpetrators were males, representing a larger proportion than reported in previous studies [18]. Data on the prevalence of IPV in male-male homosexual partnerships vary strongly [30, 31]. According to an online survey [32], 7.4% of the population in Germany define themselves as lesbian, gay, bisexual or transgender (LGBT). Against this background, our data do not point at a substantially higher proportion of IPV in homosexual partnerships, but the results have to be interpreted cautiously because of the small number of cases. Nevertheless, our data indicate a higher proportion of manual strangulation, injuries of the gluteal region and sexual violence in case of a male perpetrator, which might point at differing injury patterns in cases of IPV in male-male homosexual partnerships. This finding is in line with a meta-analytic review by Archer [22], who concluded that male perpetrators are more likely to strangle or choke their victims.

In our study group, about a third of victims reported to have experienced IPV previously, but only single individuals consulted the department repeatedly. These results are in contrast to previously reported findings, indicating higher numbers of repeated IPV (49.0–81.6%) and repeated consultations in men (7.5%) [17–19]. It should be noted, however, that repeated episodes of IPV were reported more frequently by female victims than by affected males in comparative studies [18, 19].

The affect-loaded and situational nature of IPV is reflected by the diagnosis of non-instrumental blunt violence in the majority of cases in our dataset, which is consistent with previous studies [17, 33]. When instrumental violence was used, random household items were most commonly used, and in the case of weapons being used, kitchen knives were most commonly reported. These findings reflect the affective nature of violent events in IPV by usage of objects that are readily available to the perpetrator [21, 34]. As mentioned above, we observed instrumental violence more frequently in ongoing relationships, with the shared home being the location in the majority of cases, indicating a particular risk constellation for IPV in ongoing partnerships. This observation is supported by significant differences in crime location (*p* < 0.001), reported violence (*p* = 0.002) and resulting injuries (*p* = 0.018) between offences committed by current or former partners (Table 3), but not in the tools used. The frequency of instrumental violence in our collective is similar to previous findings that also indicated more frequent use of weapons and other instrumental objects in violence against men compared to female victims [19]. Regardless of the type of injuries, the upper half of the body was primarily affected. This is consistent with studies that described the face and neck region and the upper extremities as a particularly common location of injuries due to IPV [17, 20, 21, 35] with injuries on the arms and hands partially indicating an act of defence of the victim [20, 21]. In accordance with previous reports [22], we observed scratch and bite marks more often in attacks by female offenders, although this finding was not significant. However, in studies that included male and female victims of IPV, scratch and bite injuries were diagnosed more frequently in males, while females reported strangulation more frequently [18].

Due to the severity and potential danger to life, the majority of victims with sharp violence injuries were admitted to an emergency room (76.2%), while only 36.4% of victims with blunt force injuries sought medical care. In general, 35.9% and 6.6% of injured patients visited an emergency department or office-based physician, respectively. The frequency of emergency department consultation is consistent with figures from a previous survey [18]. In the same study, male victims of IPV have been reported to present to an emergency department more frequently than female victims. This observation may be explained by the greater incidence of instrumental violence against males. These data point to the important role of the health system in the treatment and care of IPV victims, which should include both medical care and advice on local support services and require knowledge to identify potential IPV victims. Due to the high number of violence-related injuries [36–39], health professionals need specialised training in the diagnosis, documentation and care of such cases, as all medical disciplines may come into contact with victims of violence [40, 41] and therefore play a key role in recognising IPV and providing appropriate care to victims. In addition, such measures can help reduce economic consequences and psychological complications of violence, such as depressive disorders or alcohol and drug use [12, 42–46].

Limitations of the present study are the retrospective data collection, a self-selection bias, because only the victims who admitted the Forensic Medical Examination Centre for Victims of Violence could be included, and the risk of a recall bias regarding victim reports. Finally, we cannot rule out false accusations by the men in individual cases, although the forensic examination results gave no reason for this assumption.

## Conclusion

In summary, our findings point to the relevance of men as victims of IPV, case group–specific risk factors, injury-dependent behaviours related to healthcare utilisation and the need to establish or strengthen specialised support services for affected men. More than half of the victims did not seek medical care, and the Forensic Medical Examination Centre for Victims of Violence was their first contact with the healthcare system after trauma, underscoring the importance of clinical-forensic services in documenting and assessing violence-related injuries. While the medical and medicolegal management of male IPV victims does not differ significantly from female victims in terms of examination and documentation, there are specifics that should be considered when counselling affected males. In addition to raising awareness of increased risk constellations, such as instrumental violence in existing relationships or assaults in the victim’s home, our data suggest that male victims of IPV may need support and encouragement to submit to forensic examination or file police reports.
